# Transcriptomic and Metabolomic Changes Reveal the Immunomodulatory Function of Casein Phosphopeptide-Selenium Chelate in Beagle Dogs

**DOI:** 10.3390/vetsci10050345

**Published:** 2023-05-12

**Authors:** Wencan Wang, Ling Xu, Yong Cao, Guo Liu, Qianru Lin, Xin Mao

**Affiliations:** 1 Chongqing Sweet Pet Products Co., Ltd., Chongqing 400000, China; 2 Guangdong Provincial Key Laboratory of Nutraceuticals and Functional Foods, College of Food Science, South China Agricultural University, Guangzhou 510642, China

**Keywords:** casein phosphopeptide-selenium chelate (CPP-Se), transcriptome, metabolome, immunomodulatory function

## Abstract

**Simple Summary:**

A previous study by our research group demonstrated that casein phosphopeptide-selenium (CPP-Se) chelate enhanced the immune system of dogs. In this study, after feeding CPP-Se to dogs for 30 days, the leukocytes and cytokines were quantified together with the analysis of blood gene expression and serum metabolites by RNA sequencing (RNA-Seq) and metabolomics, respectively. Our findings indicate that the differentially expressed genes (DEGs) and differentially expressed metabolites (DEMs) were notably enriched in immunomodulatory and amino acid metabolic pathways, respectively. These results showed that CPP-Se can enhance immunity by regulating those genes and metabolites which are involved in immune-related pathways and also provided a theoretical basis for the future use of CPP-Se in pet foods to enhance immunity.

**Abstract:**

Casein phosphopeptide-selenium chelate (CPP-Se) is an organic compound produced by the chelation of casein phosphopeptide with selenium. This compound showed the ability to modulate canine immune response in our previous study; but its effect on the peripheral blood transcriptome and serum metabolome was unknown. This study aims to reveal the potential mechanism behind the immunomodulatory function of CPP-Se. We have identified 341 differentially expressed genes (DEGs) in CPP-Se groups as compared to the control group which comprised 110 up-regulated and 231 down-regulated genes. Kyoto Encyclopedia of Genes and Genomes (KEGG) enrichment analysis found that DEGs were mainly involved in immune-related signaling pathways. Moreover, the immune-related DEGs and hub genes were identified. Similarly, metabolomics identified 53 differentially expressed metabolites (DEMs) in the CPP-Se group, of which 17 were up-regulated and 36 were down-regulated. The pathways mainly enriched by DEMs were primary bile acid biosynthesis, tryptophan metabolism, and other amino acids metabolic pathways. Combined analysis of transcriptomic and metabolomic data showed that the DEGs and DEMs were commonly enriched in fatty acid biosynthesis, pyrimidine metabolism, glutathione metabolism, and glycerolipid metabolic pathways. Taken together, our findings provided a theoretical basis for further understanding of the immunomodulatory function of CPP-Se as well as a scientific reference for the future use of CPP-Se in pet foods as a dietary supplement to modulate the immunity.

## 1. Introduction

Due to the intimate relationship between companion animals and humans, pets are receiving more and more attention for their health problems. A critical cornerstone for maintaining their health is to enhance their immunity, which is a natural and more convenient way to combat pathogenic microorganisms. It has been proven that functional bio-actives in foods can improve animals’ immunity. Casein phosphopeptide (CPP) is an active product of milk casein hydrolysis [1], which can improve calcium absorption from the gastrointestinal tract [2], prevent bone loss [3], improve the fertilization rate in vitro [4,5] and promote apoptosis of tumor cells [6]. Additionally, CPP has been proven to regulate the immune function in a positive way across a variety of species. CPP can increase intestinal IgA levels in piglets [7], serum IgG levels in pregnant sows [8], stimulate lymphocytes proliferation and immunoglobulin production in rabbits [9], and promote the proliferation of CD19+ cells and splenocytes in mice [10,11]. Selenium (Se) is also an essential substance for the animal immune system and plays a vital role in immunomodulation. Studies have found that Se enhances immunity in pigs, increases cytokines expression, and regulates the gene expression of leukocytes [12,13]. Moreover, Se can also regulate the bovine’s cellular immune response [14] and increase the genetic expression of immunomodulators in bulls’ Sertoli cells [15].

Blood is an important component of the immune system and contains a large quantity of immune cells, including cytokines and immunoglobulins. As the blood circulates throughout the body, these immunomodulators play an extremely important role in combating the invaded pathogens [16,17]. Peripheral blood mononuclear cells (PBMCs) undergo gene expression and participate in the immune regulatory network of blood and are therefore the primary cells involved in immune response. Interestingly, nine organs, including the brain, colon, heart, kidney, liver, lung, prostate, spleen, and stomach, share approximately 80% of their transcriptome with PBMCs [18]; therefore, the rapid turnover rate of PBMCs makes it possible that disease-related subtle changes within tissues may result in a differential expression of genes in blood cells [19]. Consequently, the immunological status of different organs and tissues as well as the general health status of the body can be reflected in the genetic expression of peripheral blood [16,18]. Moreover, metabolites detected by the metabolome can reflect the physiological and pathological status of the organism [20]. Therefore, RNA sequencing (RNA-Seq) and metabolomic studies are suitable methods for investigating the immune-related functions and health status of an individual [21,22]. Currently, blood RNA-Seq and serum metabolomics have been used to study disease mechanisms [23,24,25] and immunity [19,26,27,28,29,30], but there are only a few reports available related to the use of multi-omics to explore the immune function in dogs.

Our previous study initially showed that CPP-Se can increase blood cytokine production, lymphocyte proliferation and regulate the expression of immune-related genes [31]. In the present experiment, the changes in blood transcriptome and serum metabolites were analyzed to further elucidate the potential mechanism of how CPP-Se regulates the immune function.

## 2. Materials and Methods

### 2.1. Animals and Experimental Design

Twelve healthy, adult Beagle dogs (1.5 years old, mean 13 kg weight) were used in this experiment and were divided into two groups i.e., a control and a treatment group. All the animals were fed twice a day (9:00 am and 3:00 pm) with maintenance food (Jiangsu Xietong Inc., Nanjing, China). Two hours after being fed the maintenance food, the control and experimental groups were respectively fed 30 g of the control snack without CPP-Se and 30 g of the test snack containing 0.03% CPP-Se (Chongqing Sweet Pet Products Co., Ltd., Chongqing, China). The dogs were acclimatized for 7 days with the snacks before the start of the experiment and provided with water ad libitum during the 30-day experiment.

**Figure 1:**
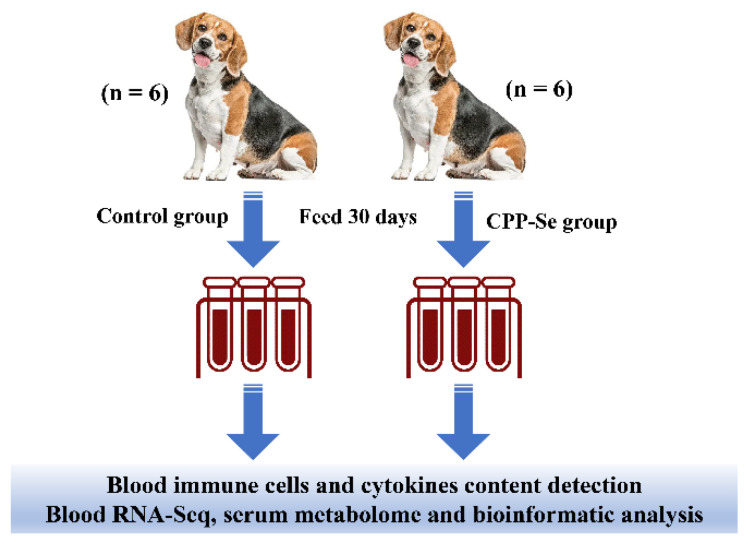
Experimental design.

The CPP-Se was provided by the College of Food Science, South China Agricultural University. All the procedures were approved by the Institutional Animal Care and Use Committee of South China Agriculture University (Permit Number: SCAU-AEC-2010-0416). The schematic diagram of the experimental design is shown in Figure 1.

### 2.2. Hematology Routine and Serum Cytokines Concentration Detection

Canine peripheral blood was collected according to the previous method [31]. Briefly, 1 mL of blood was collected from the saphenous vein, and added to an EDTA-K_2_ anticoagulation tube, gently mixed and stored at 4 °C for transport to the laboratory, followed immediately by a routine blood analysis (Mindray, Shenzhen, China) to measure the blood leukocyte. Serum was collected according to Zheng’s method [32]. Briefly, 2 mL of canine peripheral blood was collected from the saphenous vein and added to a serum separator tube which was left at room temperature for 1 h and then centrifuged at 3000 rpm for 10 min at 4 °C. The serum was then divided into 1.5 mL EP tubes and stored at −20 °C for cytokines detection and metabolite analysis. The concentration of serum IL-4, IL-6, IgM, IgG and IFN-γ was detected according to the instructions given with ELISA kits (FANKEL Industrial Co., Ltd., Fankew, Shanghai, China).

### 2.3. RNA-Seq and Data Analysis

#### 2.3.1. RNA Library Construction and Sequencing

After whole blood collection, the RNA was extracted by Trizol LS (Aidlab Biotechnologies, Beijing, China) reagent and assayed for integrity and total RNA concentration using an Agilent 2100 bioanalyzer (Agilent, Santa Clara, CA, USA). The poly-A mRNAs were enriched by Oligo (dT) magnetic beads and then the divalent cations were used to randomly break mRNAs in an NEB fragmentation buffer, followed by library construction using the NEBNext^®^ Ultra™ RNA Library Prep Kit for Illumina^®^ (NEB, Ipswich, MA, USA); index codes were added to attribute sequences to each sample. After library construction, initial quantification was performed using a Qubit 2.0 Fluorometer, followed by dilution of the library to 1.5 ng/μL and the detection of the insert size of the library using an Agilent 2100 bioanalyzer. RT-qPCR was performed to accurately quantify the effective concentration of the library to ensure the libraries’ quality. T RNA sequencing was then performed by the Novogene Bioinformatics Institute (Novogene, Beijing, China) for the paired-end sequencing on the Illumina NovaSeq 6000 platform (Illumina, Inc., San Diego, CA, USA).

#### 2.3.2. Quality Control and Differentially Expressed Genes (DEGs) Identification

The raw data generated by RNA-Seq contain few sequencing junctions or low-quality reads. Therefore, in order to obtain high-quality data for downstream analyses, reads with junctions and containing N (N means the base information is uncertain) as well as low-quality reads were filtered together with the calculation of Q20, Q30 and GC contents. HISAT2 (v 2.0.5) was used to construct an index of the reference genome and clean reads were aligned to the reference genome (https://ftp.ensembl.org/pub/release-100/fasta/canis_lupus_familiaris/, accessed on 13 June 2022). The assembly of reads aligned to the reference genome was prepared using StringTie (v 1.3.3b) [33]. Based on the gene length and the number of reads mapped to this related gene, the FPKM of each gene was calculated. The heatmap was constructed using FPKM and the differential expression between the two groups was analyzed using DESeq2 software (v 1.20.0). Genes with a *p*-adj < 0.05 and |Fold Change| ≥ 1.5 were considered as DEGs. The RNA-Seq data were deposited in the Gene Expression Omnibus (GEO) datasets on NCBI.

#### 2.3.3. Functional Enrichment Analysis of DEGs

Gene Ontology (GO) and Kyoto Encyclopedia of Gene and Genome (KEGG) enrichment analysis of DEGs was performed using the clusterProfiler R package (v 3.8.1) [34] and *p* < 0.05 was considered as significant.

### 2.4. Screening for Immune-Related Genes

Immune-related genes of DEGs were screened using the ImmPort database (https://www.immport.org/home, accessed on 4 February 2023) and obtained from genes’ lists (immunologically relevant lists of genes curated with functions and Gene Ontology terms) [35].

### 2.5. Protein–Protein Interaction (PPI) Network Construction and Hub Genes Analysis of DEGs

The PPI networks were constructed using the online String database (https://www.string-db.org/, accessed on 4 February 2023) and an interaction with a combined score of ≥ 0.4 was considered significant. Hub genes analysis was then performed using the cyto-Hubba application (v 0.1) from Cytoscape (v 3.9.0) based on the degree method. KEGG enrichment analysis of hub genes was performed using the DAVID online database (https://david.ncifcrf.gov/conversion.jsp, accessed on 4 February 2023).

### 2.6. Untargeted Metabolomics and Data Analysis

#### 2.6.1. Metabolites Extraction

100 μL of serum was resuspended with pre-chilled methanol (80%), incubated on ice for 5 min and then centrifuged at 15,000× *g* for 20 min at 4 °C. The required amount of supernatant was diluted with LC-MS grade water to a final concentration of 53% methanol. The supernatant was then transferred to new EP tubes and then centrifuged at 15,000× *g* for 20 min at 4 °C, and finally transferred to the LC-MS/MS system for analysis [36].

#### 2.6.2. UHPLC-MS/MS Analysis

A Vanquish UHPLC system (Thermo Fisher, Waltham, MA, USA) connected to an Orbitrap Q Exactive^TM^ HF mass spectrometer (Thermo Fisher, Waltham, MA, USA) was used for UHPLC-MS/MS analysis. Serum was injected into a Hypersil Gold column using a 12-min linear gradient at a flow rate of 0.2 mL/min. The eluents used were 0.1% FA in water (eluent A) and methanol (eluent B) for the positive polarity mode and 5 mM ammonium acetate (eluent A) and methanol (eluent B) for the negative polarity mode (eluent B). The settings were as follows: (1) Spray voltage: 3.5 kV, (2) Capillary temperature: 320 °C, (3) Sheath gas flow rate: 35 psi and aux gas flow rate: 10 L/min, (4) S-lens RF level: 60, and (5) aux gas heater temperature: 350 °C.

#### 2.6.3. Data Processing and Metabolite Identification

A Compound Discoverer 3.1 (Thermo Fisher, Waltham, MA, USA) was used to process the raw data files and to perform peak alignment and selection as well as metabolite quantitation. Peak intensities were then adjusted to reflect the total spectral intensity. According to the additive ions, molecular ion peaks and fragmented ions, the normalized data were used to predict the molecular formula of the metabolites. Subsequently, the actual data, including qualitative and relative quantitative results, were obtained using mzCloud (https://www.mzcloud.org/, accessed on 5 February 2023), mzVault, and the MassList database.

#### 2.6.4. Data Analysis

The metabolites were annotated using the KEGG database (https://www.genome.jp/kegg/pathway.html, accessed on 10 February 2023), HMDB database (https://hmdb.ca/metabolites, accessed on 10 February 2023), and LIPIDMaps database (http://www.lipidmaps.org/, accessed on 10 February 2023). Principal component analysis (PCA) and partial least squares discriminant analysis (PLS-DA) were conducted using metaX [37]. Univariate analysis (*t*-test) was used to calculate the statistical significance. The metabolites with VIP > 1 and *p* < 0.05 and |fold change|≥ 2 were considered as differentially expressed metabolites (DEMs). The DEMs based on log_2_ (fold change) and -log10 (*p*-value) were presented using volcano plots. The data were normalized using the z-scores of the DEMs intensity area for clustering heat maps, and a pheatmap tool was used to decipher the results. The correlation between DEMs was examined, and the R language was used to assess the significance of the correlation (*p* < 0.05). The KEGG database was used to assess the pathways enriched by the DEMs, and an enrichment of *p* < 0.05 was considered as significant.

### 2.7. Combined Analysis of RNA-Seq and Metabolome Data

Correlations between RNA-seq and metabolomic data were assessed by Pearson correlation analysis. According to the correlation coefficients, the interaction network between DEMs and hub genes was plotted using Cytoscape. Moreover, the common pathways between DEGs and DEMs were analyzed by an online MetaboAnalyst 5.0 tool (https://www.metaboanalyst.ca, accessed on 15 February 2023).

### 2.8. Total RNA Extraction, cDNA Synthesis and RT-qPCR

Whole blood RNA was extracted with Trizol LS (Aidlab Biotechnologies, Beijing, China) reagent. The Nano Pro 2010 (DHS Life Science and Technology, Tianjin, China) was used to determine the concentration and quality of RNA. The cDNA was prepared with ABScript III RT Master Mix kit (ABclonal, Wuhan, China) according to the manufacturer’s instructions. The prepared cDNA was stored at −20 °C and RT-qPCR was performed using 2× Universal SYBR Green Fast qPCR Mix (ABclonal, Wuhan, China) on an RT-qPCR detection system (Bioer Technology, Hangzhou, China). Briefly, for relative mRNA expression assays, we used a 10 µL system containing 5 µL SYBR Green I Premix (ABclonal, Wuhan, China), 0.5 µL each of forward and reverse primers (Supplementary Table S1), 1 µL cDNA and 3 µL RNase-free water. Relative mRNA expression levels were calculated using the 2^−∆∆CT^ method [38].

### 2.9. Statistical Analysis

All data were tested for normality and homogeneity of variance prior to statistical analysis. Data between the two groups were analyzed by Student *t*-test and a value of *p* < 0.05 was considered as statistically significant; experimental results are expressed as mean ± standard error (SEM).

## 3. Results

### 3.1. Evaluation of Immune-Related Parameters

**Figure 2:**
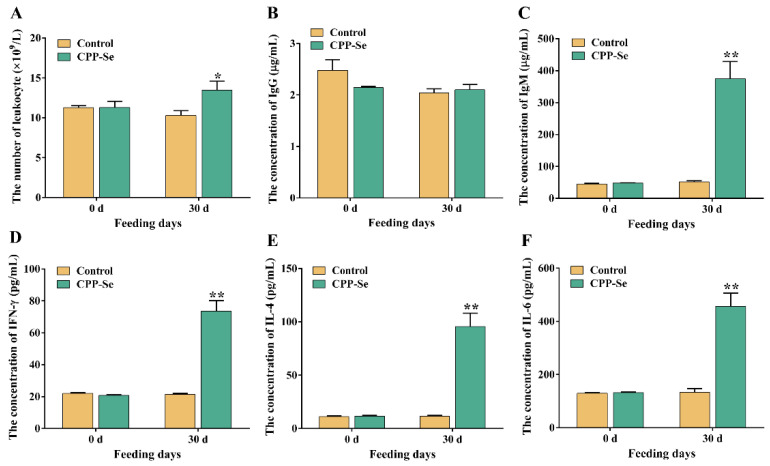
The number of blood leukocytes (**A**) and the levels of serum IgM (**B**), IgG (**C**), IFN-γ (**D**), IL-4 (**E**) and IL-6 (**F**) between control and CPP-Se groups. “*” indicates *p* < 0.05 and “**” indicates *p* < 0.01.

The number of blood leukocytes in the CPP-Se group increased significantly as compared to the control group after 30 days of CPP-Se feeding (Figure 2A, *p* < 0.05). Serum IgG levels did not change noticeably after feeding CPP-Se (Figure 2B, *p* > 0.05), but IgM levels increased significantly (Figure 2C, *p* < 0.01). Additionally, the contents of IFN-γ, IL-4 and IL-6 were increased significantly in the CPP-Se group compared to the control group (Figure 2D,F, *p* < 0.01).

### 3.2. Summary of RNA-Seq Data

**Figure 3:**
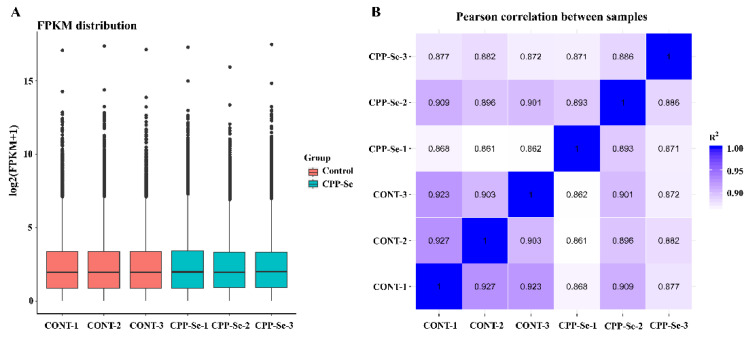
The gene expression boxplot (**A**) and Pearson correlation analysis (**B**) of all samples.

After we had filtered the raw data and checked the error rate, we obtained a total of 39.98 Gb of clean reads data from all the samples. The error rate < 0.03, Q30 > 92.77%, total mapped ratio went from 94.98% to 96.04% and the GC content from 57.16% to 57.85%, indicating that these were high-quality reads (Supplementary Table S2). Moreover, the boxplot of genes’ FPKM in each sample showed the overall gene expression pattern of the different sample (Figure 3A). Correlation analysis showed that the R^2^ > 0.8 between biological replicates within groups and between different samples (Figure 3B), and these results suggested that the sequencing data are reliable and can be further analyzed.

### 3.3. DEGs and Functional Enrichment Analysis

**Figure 4:**
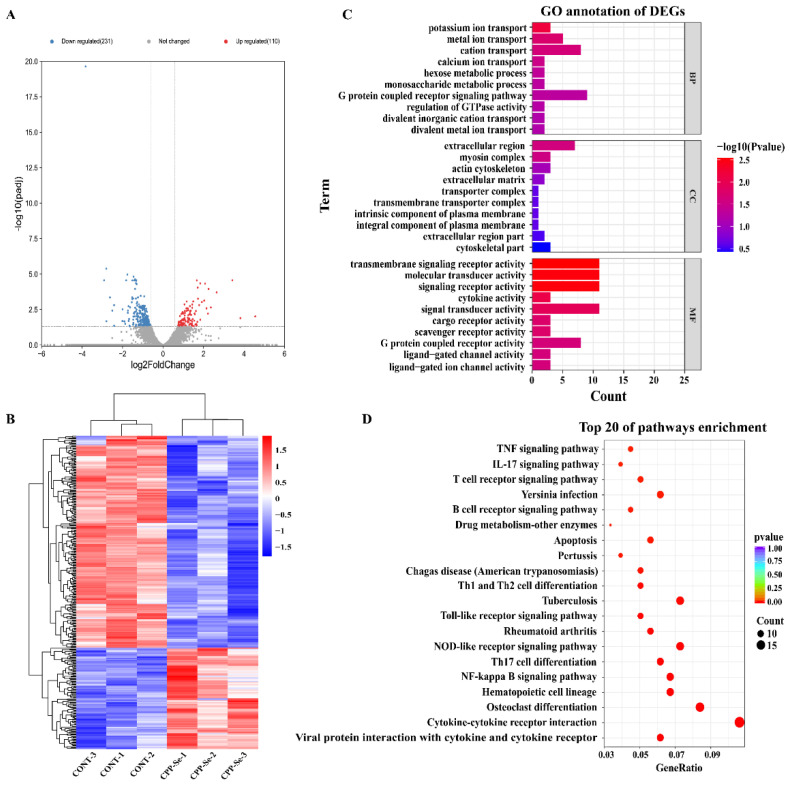
Analysis of DEGs. Volcano plot (**A**) and hierarchical cluster analysis (**B**) of DEGs in control and CPP-Se groups. (**C**) GO analysis. (**D**) KEGG enrichment analysis.

Compared with the control group, 110 significantly up-regulated and 231 down-regulated genes were identified in the CPP-Se group (Figure 4A, Supplementary Table S3). Hierarchical clustering of all the DEGs (Figure 4B) was used to show the gene expression pattern and the top 20 significantly up- and down-regulated genes are shown in Supplementary Tables S4 and S5, respectively.

The 341 DEGs were subjected to GO analysis, including the biological process (BP), cellular component (CC) and molecular function (MF). In our study, a total of 444 GO terms was found to be enriched (Supplementary Table S6). The top 10 terms of each category are shown in Figure 4C. The important BP terms were ion transport and the metabolic process. Similarly, the predominant enriched CC terms were the extracellular region, myosin complex, and actin cytoskeleton. The most significant MF terms were the transmembrane signaling receptor activity, signaling receptor activity, molecular transducer activity, and cytokine activity. Moreover, KEGG analysis revealed a total of 246 enriched pathways (Supplementary Table S7). Among the top 20 pathways, most are related to immune responses, such as the cytokine–cytokine receptor interaction signaling pathway, TCR signaling pathway, TNF signaling pathway, B cell receptor signaling pathway, NF-kappa B signaling pathway, and Toll-like receptor signaling pathway (Figure 4D).

### 3.4. Immune-Related Genes and Hub Genes Analysis of DEGs

**Figure 5:**
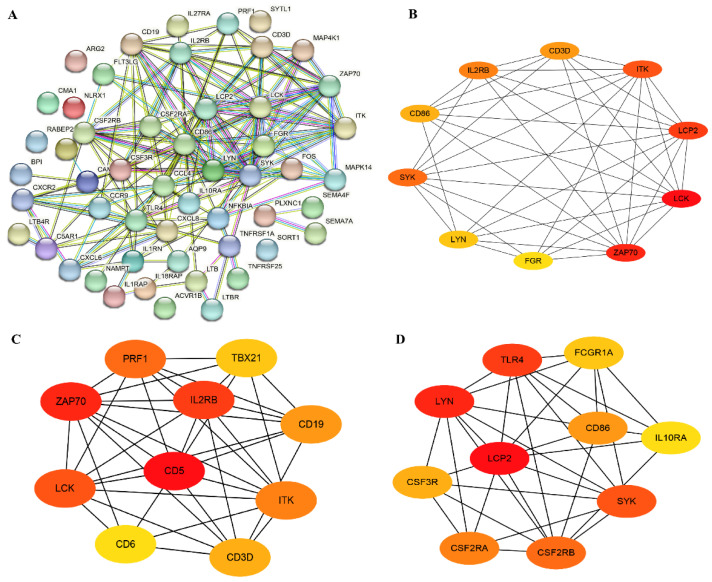
Immune-related genes and hub genes analysis of DEGs. (**A**) The PPI network of immune-related genes. (**B**) The hub genes analysis of immune-related genes. (**C**) The hub genes screening of all up-regulated DEGs. (**D**) The hub genes screening of all down-regulated DEGs.

A total of 52 immune-related genes was screened from DEGs, out of which 19 were found to be up-regulated and 33 were down-regulated (Supplementary Table S8); the top 10 up-/down-regulated DEGs were selected for display according to the log_2_FC in Supplementary Table S9. Most of these DEGs are associated with cytokine–cytokine receptor mediated immunomodulatory pathways, such as SEMA4F, PRF1, LTB, IL18RAP, IL1RAP and *TLR4*. Furthermore, we performed PPI and hub genes analysis of these immune-related DEGs, and found that *ZAP70*, *LCK* and *LCP2* were hub genes based on the node score (Figure 5A,B).

PPI networks of all up- and down-regulated genes were constructed, and the hub genes were screened. The top 10 most significant up-regulated hub genes were IL2RB, CD5, PRF1, TBX21, *ZAP70*, CD19, *LCK*, CD6, CD3D and ITK (Figure 5C), and the top 10 most significant down-regulated hub genes were *TLR4*, FCGR1A, CD86, *LCP2*, LYN, IL10RA, CSF3R, SYK, CSF2RA and CSF2RB (Figure 5D). Among them, the *ZAP70* and LCK were immune-related up-regulated DEGs. Furthermore, pathway analysis of these 20 hub genes showed that several immune-related pathways were enriched, including the cytokine–cytokine receptor interaction pathway, the NF-kappa B signaling pathway, and the TCR signaling pathway (Supplementary Table S10).

### 3.5. Analysis of DEGs Related to Cytokine–Cytokine Receptor Interaction Signaling Pathway and TCR Signaling Pathway

**Figure 6:**
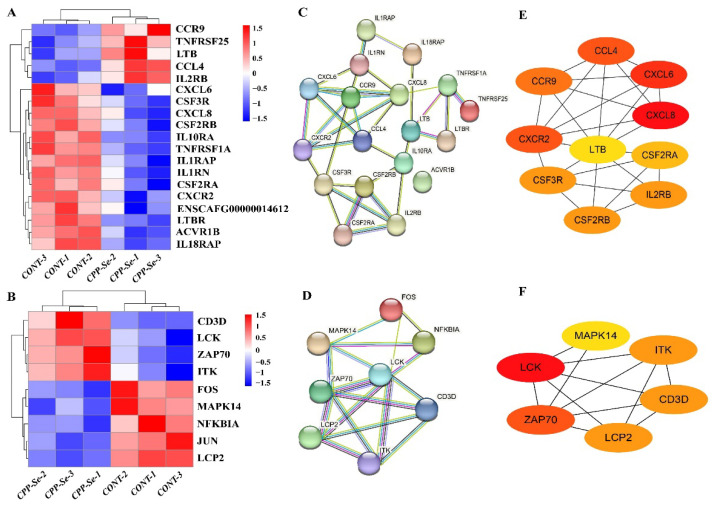
Analysis of DEGs related to cytokine–cytokine receptor interaction signaling pathway and TCR signaling pathway. (**A**) DEGs involved in cytokine–cytokine receptor interaction signaling pathway. (**B**) DEGs involved in TCR signaling pathway. (**C**) PPI network of DEGs in cytokine–cytokine receptor interaction signaling pathway. (**D**) PPI network of DEGs in TCR signaling pathway. (**E**) Hub genes analysis of DEGs in cytokine–cytokine receptor interaction signaling pathway. (**F**) Hub genes analysis of DEGs in TCR signaling pathway.

Cytokines and T cells are the vital components of the immune system that keep the body in a normal physiological state. KEGG analysis indicated that the highest number of DEGs was involved in cytokine–cytokine receptor interaction signaling pathways, and the immune-related genes were mostly associated with cytokines and cytokine receptors. In addition, the immune-related genes *LCK*, *ZAP70* and *LCP2* were co-enriched in the TCR signaling pathway. For further verification, DEGs in the cytokine–cytokine receptor interaction signaling pathway and the TCR signaling pathway were analyzed. In total, 175 genes were found to be enriched in the cytokine–cytokine receptor interaction signaling pathway, including 19 DEGs (5 up-regulated and 14 down-regulated, Figure 6A). Moreover, 115 genes were associated with the TCR signaling pathway, including 9 DEGs (4 up-regulated and 5 down-regulated, Figure 6B). PPI networks were constructed from the DEGs (Figure 6C,D) and the hub genes were identified. According to the node degree, *CCL4*, CXCR2, CXCL6, *CXCL8* and *CCR9* were important hub genes present in the cytokine–cytokine receptor interaction signaling pathway (Figure 6E), while the other hub genes, i.e., *LCK* and *ZAP70*, present in the TCR signaling pathway are also related to the immune system (Figure 6F).

### 3.6. Untargeted Metabolomics and DEMs Analyses

**Figure 7:**
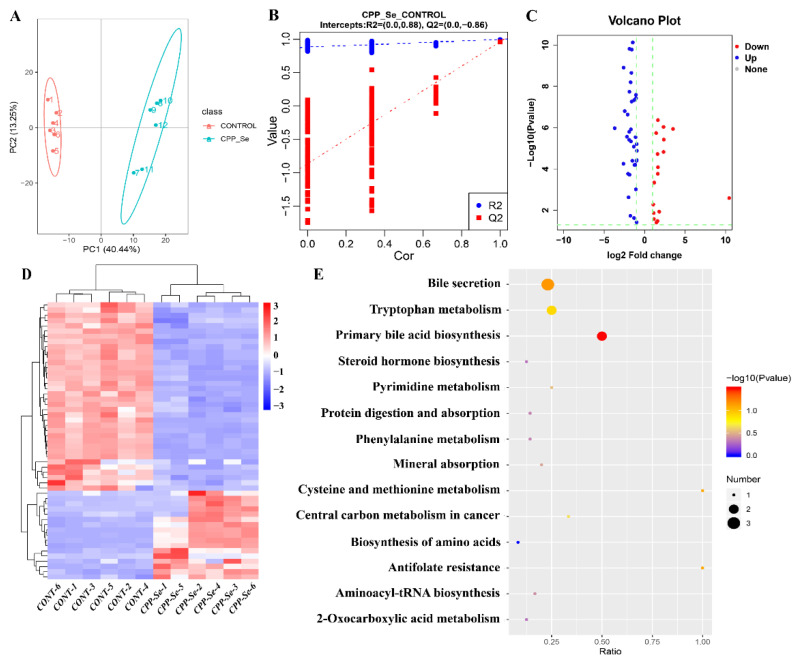
Metabolome analysis of serum. (**A**) PCA analysis of metabolites. (**B**) OPLS-DA score plot of metabolites. (**C**) Volcano plot of DEMs. (**D**) Clustering heatmap of DEMs. (**E**) KEGG pathways analysis of DEMs.

The dogs’ serum metabolic changes that resulted from CPP-Se supplementation were explored and the DEMs were identified by metabolome analysis. The PCA and OPLS-DA showed a significant difference in metabolic profiles between the CPP-Se and control groups (Figure 7A,B). A total of 53 DEMs was identified in the CPP-Se group compared to the control group, including 17 up-regulated and 36 down-regulated metabolites (Figure 7C, Supplementary Table S11). The hierarchical clustering of DEMs is shown in Figure 7D. KEGG enrichment analysis showed that the pathways enriched by DEMs were bile secretion, primary bile acid biosynthesis, tryptophan metabolism and other amino acids metabolic pathways (Figure 7E).

### 3.7. Combined Analysis of Transcriptomics and Metabolomics

**Figure 8:**
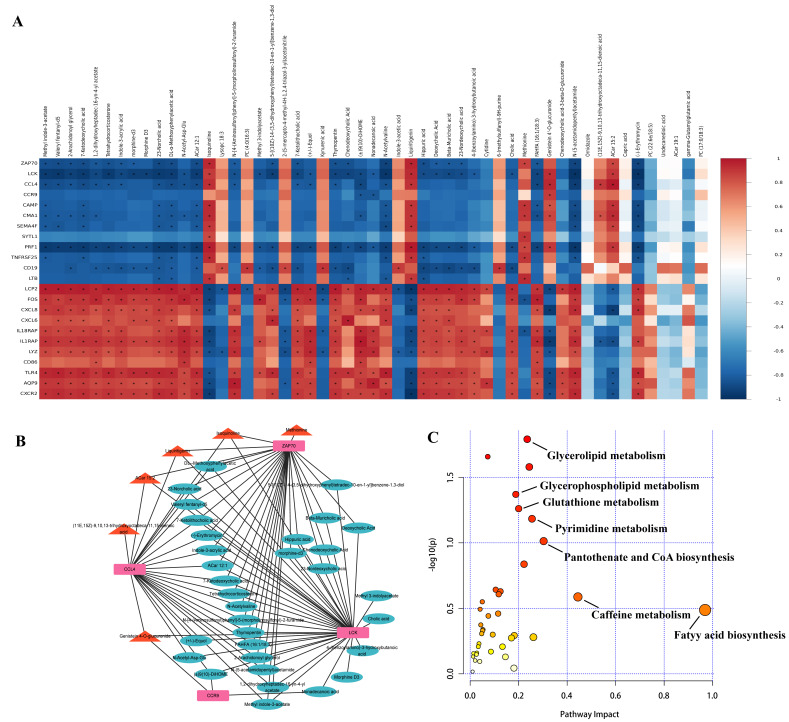
Combined analysis between transcriptome and metabolome data. (**A**) Pearson correlation analysis of DEGs and DEMs. The red and blue colors represent positive and negative correlations, respectively. “*” indicates *p* < 0.05. (**B**) Interaction network of hub genes and DEMs. Pink rectangles represent up-regulated hub genes, red triangles represent up-regulated DEMs that are significantly positively associated with hub genes, and light blue ellipses represent down-regulated DEMs that are significantly negatively associated with hub genes. (**C**) Common pathways between DEGs and DEMs.

To reveal the correlation between transcriptomics and metabolomics, the correlations between DEGs (top 10 up-/down-regulated immune-related DEGs and hub genes *ZAP70*, *LCK*, *CCL4*, *CCR9*) and DEMs were calculated by using the Pearson correlation analysis, where correlation coefficients values >0 and <0 were considered as positive and negative correlations, respectively (Figure 8A). Further, correlation coefficients value of >0.8 and *p* < 0.5 were considered as strong correlations, and the interaction networks between these four hub genes and DEMs were plotted by Cytoscape. The results showed a significant positive correlation between methionine, isoquinoline, liquiritigenin, ACar 15:2 (11E,15Z)-9,10,13-trihydroxyoctadeca-11,15-dienoic acid, genistein 4-O-glucuronide and hub genes, suggesting that these DEMs may be synergistically involved with hub genes in immune regulation (Figure 8B). Moreover, KEGG analysis showed the pathways which were commonly enriched by DEGs and DEMs were fatty acid biosynthesis, glycerolipid metabolism, glycerophospholipid metabolism, pyrimidine metabolism, etc (Figure 8C).

### 3.8. RT-qPCR Validation

**Figure 9:**
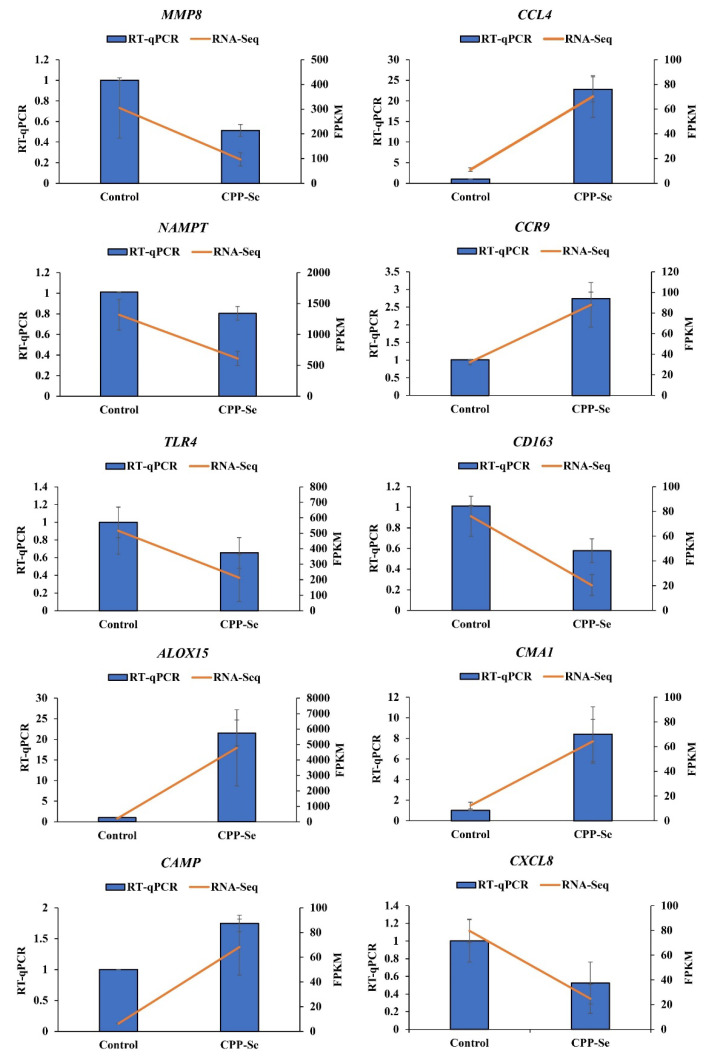
RT-qPCR validation.

We randomly selected 10 DEGs (*MMP8*, *CCL4*, *NAMPT*, *CCR9*, *TLR4*, *CD163*, *ALOX15*, *CMA1*, *CAMP* and *CXCL8*) for RT-qPCR detection, and the results showed that the expression trends of these DEGs were consistent with the sequencing data (Figure 9), further showing that our sequencing data are reliable.

## 4. Discussion

Animals with better immunity can fend off disease-causing microorganisms and stay healthy. In our previous study, we observed that CPP-Se could increase some immunological markers in dogs, including lymphocyte counts and cytokine levels [31]. In this research, we further investigated the effect of CPP-Se on canine immune function by blood RNA-Seq and metabolome analysis. First, blood leukocytes, serum cytokines and immunoglobulin levels were assessed after CPP-Se supplementation. Leukocytes and cytokines are important components of the blood immune system and play a vital role in combating infection and inflammation in animals’ bodies [39,40]. Our results showed that CPP-Se was effective in increasing leukocyte counts and cytokine levels, which are consistent with previous studies. Transcriptome analysis indicated that 341 DEGs were identified in the CPP-Se group compared to the control group and these DEGs were significantly enriched in several immunomodulatory pathways, suggesting that CPP-Se affects the blood gene expression profile of dogs.

The cytokine–cytokine receptor interaction signaling pathway is an essential immune related pathway which is involved in the regulation of disease processes in many species [28,41,42,43,44]. Our enrichment analysis revealed that the cytokine–cytokine receptor interaction signaling pathway was significantly enriched by DEGs. Ye et al. demonstrated that specifically salmonella or reproductive respiratory syndrome virus infection significantly up-regulate the cytokine mRNAs expression in pigs, and that DEGs in the blood were also enriched in the cytokine–cytokine receptor interaction signaling pathway, demonstrating that specific factors in this pathway are involved in blood immune system regulation and reflect the health status of the organism [29]. Similarly, Qian et al. demonstrated that cytokine–cytokine receptor interaction plays a critical role in the clearance of the respiratory syncytial virus in vivo [45]. In the present study, the cytokine–cytokine receptor interaction signaling pathway was the most enriched for DEGs, and cytokines or cytokine receptors were encoded by many immune-related DEGs. In this pathway, *CCL4* and *CCR9* are up-regulated hub genes encoding chemokines and chemokine receptors, respectively. An important function of chemokines is to direct immune cells to the site of inflammation and thereby defend and eliminate the pathogenic microbes [46,47]. *CCL4* is a member of the CC chemokine family and binds to its receptors to regulate immune cell migration and stimulate T cell activation and differentiation [48,49]. In addition, *CCL4* has been found to have antimicrobial activity [50]. Our study showed that CPP-Se increased *CCL4* expression and presumably increased the resistance to pathogenic infection. Similarly, *CCR9* belongs to the chemokine receptor family and is mainly expressed on immature T cells together with neutrophils and mononuclear macrophages [51]. The role of *CCR9* in inflammatory diseases is currently being investigated. Huang et al. found a significant increase in *CCR9* in mice suffering with myocardial infarction, suggesting that *CCR9* plays an important role in inflammatory cells infiltration [52]. In addition, *CCR9*+ T cells and plasmacytoid dendritic cells can reach the small intestine under the guidance of ligands, suggesting that *CCR9* plays a vital role in regulating the intestinal immune response [53,54]. Wurbel et al. found that *CCR9* knockout mice were more susceptible to colitis caused by infectious agents and recovered more slowly, and the colonic mucosa was found to harbor a large number of macrophages and inflammatory cytokines, suggesting that *CCR9* may be able to regulate the response of colonic immune cells [55]. Our results showed that CPP-Se could increase *CCR9* expression, indicating that the immune system of dogs is enhanced to some extent. Additionally, the immune function of the gut may be concomitantly improved, but this needs to be demonstrated by more in-depth studies.

The sequencing data showed that CPP-Se significantly increased the expression of *LCK* and *ZAP70* genes, which are enriched in the TCR signaling pathway. Importantly, *LCK* and *ZAP70* are also immune-related hub genes. The TCR signaling pathway is a critical pathway for the recognition of external pathogenic microorganisms [56]. In the TCR signaling pathway, the phosphorylation of *LCK* recruits and activates *ZAP70*, which mediates T cell maturation and activation [57]. Mutation of *ZAP70* was found to cause T cell-related immune deficiency in zebrafish [58]. In addition, the deletion of *ZAP70* reduced the number of *CD8+* T cells, resulting in a loss of ability to activate the TCR pathway, T cell proliferation, and cytokine expression [59]. Wang et al. concluded that the expressions of *LCK* and *ZAP70* were significantly downregulated in the submandibular gland of juvenile goats compared to adult goats, and because the immune function in the submandibular glands of goats gradually decreases with growth, this suggests that there is a positive correlation between *LCK, ZAP70*, and immune function [60]. Certain bio-actives, such as amino acids, can affect immunity by modulating the proliferative capacity of T cells [61]. Wu et al. found that asparagine phosphorylates *LCK*, which activates the TCR signaling pathway and promotes T cell activation, resulting in increased responses to pathogens and tumor cells, ultimately leads to improved immunity [62]. Du et al. illustrated that hydrolytic amino acids increased *ZAP70* expression in mouse lymphocytes, accompanied by lymphocyte proliferation and cytokine production; this demonstrates that *ZAP70* can activate lymphocytes and improve immune function [63]. Our results showed that CPP-Se increased the mRNA expression of *LCK* and *ZAP70*, suggesting that CPP-Se may enhance the immunity of dogs by promoting the immune function of T cells and the TCR signaling pathways.

Our metabolomic data showed that the pathways highly enriched by DEMs included several amino acids metabolism pathways, such as tryptophan, phenylalanine, cysteine, and methionine amino acid metabolism pathways. Studies have shown that amino acids metabolism is closely associated with immune response [64,65]. Kynurenic acid (KYNA), a metabolic product of tryptophan, was found to stimulate the expression of IL-6 in human breast cancer cells as well as cytokine production in mouse splenocytes [66,67]. Barth et al. showed that KYNA exerts its immunomodulatory function by activating neutrophils and recruiting leukocytes [68]. In addition, KYNA decreased TNF and nitric oxide levels in the serum of mice treated with LPS, and increased survival rates after LPS infection [69].

Correlation analysis showed that methionine, isoquinoline and liquiritigenin were positively correlated with *ZAP70*, *LCK* and *CCL4*. Methionine is considered to be an important amino acid that affects the immune function of the body. Numerous studies have demonstrated that methionine enhances the cellular immune response [70], stimulates leukocytes migration [71], promotes the development of bursa [72] and increases the differentiation of thymic T cells [73] in poultry. Soder et al. discovered that methionine stimulates the proliferation of peripheral blood lymphocytes in dairy cows [74]. During in vitro pathogen infection, Zhou et al. observed an increase in granulocytic phagocytosis in cows fed a supplemented fodder with methionine, indicating that methionine may enhance the immune system [75]. Moreover, reduced lymphocyte counts, increased susceptibility to bacterial infection and a lower responsiveness to mitogens are all consequences of methionine restriction in mice [76]. Isoquinoline is a class of alkaloids with anti-inflammatory activity and has been proven to inhibit LPS-induced inflammation and apoptosis in mouse cardiomyocytes [77]. Pickler et al. found that the sanguinarine, a major component of isoquinoline alkaloids, reduced the detection rate of bacteria in the cecum and increased the proportion CD4+ and CD8α+ cells in the blood in a model of salmonella enteritis [78]. Liquiritigenin, a flavonoid extracted from licorice, has been illustrated to increase the concentration of cAMP in dendritic and T cells, thereby regulating cytokine production and exerting anti-inflammatory effects [79]. Treatment of mouse macrophages with liquiritigenin suppressed the LPS-induced NF-κB DNA binding activity and inhibited the LPS-stimulated production of iNOS proteins. Furthermore, liquiritigenin reduced the levels of TNF-α, IL-1β and IL-6 in macrophages exposed to LPS, suggesting that liquiritigenin has anti-inflammatory effects [80]. In our study, KYNA, methionine, isoquinoline, and liquiritigenin were notably increased after CPP-Se feeding, suggesting that these metabolites may work in concert with immune-related hub genes to exert an immunomodulatory function after CPP-Se supplementation.

In this study, the combined analysis showed that the pathways which are commonly enriched by DEGs and DEMs, such as glycerolipid metabolism, pyrimidine metabolism, and glutathione metabolism, need further attention, as some reports have indicated that these pathways are also involved in immune regulation [81,82,83].

## 5. Conclusions

In conclusion, we used RNA-Seq and metabolomic analysis for the first time to explore changes in the blood transcriptome and serum metabolome of dogs fed CPP-Se. The results showed that CPP-Se can regulate the expression of immune-related hub genes, such as *CCL4, CCR9, ZAP70*, and *LCK*, which play critical roles in the immune system. KEGG analysis demonstrated that DEGs were enriched in several immune regulatory pathways, out of which the cytokine–cytokine receptor interaction signaling pathway and the TCR signaling pathway were the significant ones, indicating their important functions in the regulation of the canine immune system by CPP-Se. In addition, the metabolomic data indicated that the amino acids metabolism was mainly affected by CPP-Se, and the significant positive correlations among methionine, isoquinoline, liquiritigenin and hub genes were identified, and these metabolites were closely linked with immune functions. As a consequence, we have elucidated the underlying mechanism by which CPP-Se exerts its immunomodulatory effects, although this requires further research to demonstrate; however, the results of our present study still bring prospective value to the addition of CPP-Se in pets’ routine diet to enhance immunity.

## Data Availability

The RNA-seq raw data are available at NCBI GEO (Gene Expression. Omnibus) databases under the accession number GSE222161.The metabolomic data are available in the OMIX, China National Center for Bioinformation/Beijing Institute of Genomics, Chinese Academy of Sciences (https://ngdc.cncb.ac.cn/omix, accession no: OMIX003027, accessed on 10 February 2023).

## Supplementary Materials

The following supporting information can be downloaded at: https://www.mdpi.com/article/10.3390/vetsci10050345/s1, Table S1: The primers sequences information. Table S2: Overview of RNA-Seq data in control and CPP-Se groups. Table S3: The DEGs between CPP-Se and control groups. Table S4: Top 20 up-regulated DEGs between the CPP-Se and control group. Table S5: Top 20 down-regulated DEGs between the CPP-Se and control group. Table S6: The GO enrichment analysis of DEGs. Table S7: The KEGG enrichment analysis of DEGs. Table S8: The list of immune-related genes; Table S9: Top 10 up/down-regulated immune-related DEGs. Table S10: The KEGG pathways analysis of 20 hub genes. Table S11: The DEMs between CPP-Se and control groups.
